# The Odyssey of Dental Anxiety: From Prehistory to the Present. A Narrative Review

**DOI:** 10.3389/fpsyg.2017.01155

**Published:** 2017-07-11

**Authors:** Enrico Facco, Gastone Zanette

**Affiliations:** ^1^Studium Patavinum, University of Padua Padua, Italy; ^2^Franco Granone Institute – Italian Center of Clinical & Experimental Hypnosis (CIICS) Turin, Italy; ^3^Chair of Dental Anesthesia, Department of Neurosciences, University of Padua Padua, Italy

**Keywords:** anxiety, behavioral, conscious sedation, dental health, dentistry, depression, fear, hypnosis

## Abstract

Dental anxiety (DA) can be considered as a universal phenomenon with a high prevalence worldwide; DA and pain are also the main causes for medical emergencies in the dental office, so their prevention is an essential part of patient safety and overall quality of care. Being DA and its consequences closely related to the *fight-or-flight* reaction, it seems reasonable to argue that the odyssey of DA began way back in the distant past, and has since probably evolved in parallel with the development of *fight-or-flight* reactions, implicit memory and knowledge, and ultimately consciousness. Basic emotions are related to survival functions in an inseparable psychosomatic unity that enable an immediate response to critical situations rather than generating knowledge, which is why many anxious patients are unaware of the cause of their anxiety. Archeological findings suggest that humans have been surprisingly skillful and knowledgeable since prehistory. Neanderthals used medicinal plants; and relics of dental tools bear witness to a kind of Neolithic proto-dentistry. In the two millennia BC, Egyptian and Greek physicians used both plants (such as *papaver somniferum*) and incubation (a forerunner of modern hypnosis, e.g., in the sleep temples dedicated to Asclepius) in the attempt to provide some form of therapy and painless surgery, whereas modern scientific medicine strongly understated the role of subjectivity and mind-body approaches until recently. DA has a wide range of causes and its management is far from being a matter of identifying the ideal sedative drug. A patient's proper management must include assessing his/her dental anxiety, ensuring good communications, and providing information (iatrosedation), effective local anesthesia, hypnosis, and/or a wise use of sedative drugs where necessary. Any weak link in this chain can cause avoidable suffering, mistrust, and emergencies, as well as having lifelong psychological consequences. Iatrosedation and hypnosis are no less relevant than drugs and should be considered as primary tools for the management of DA. Unlike pharmacological sedation, they allow to help patients cope with the dental procedure and also overcome their anxiety: achieving the latter may enable them to face future dental care autonomously, whereas pharmacological sedation can only afford a transient respite.

## Introduction

The concept of anxiety (like the core concept of consciousness and all the terms related to subjectivity) has several possible meanings, and may thus give rise to some uncertainty and ambiguity (Facco et al., 2017). The generally available dictionary definitions are unsatisfactory. For instance, the Merriam-Webster dictionary^1^ defines anxiety as an “*Apprehensive uneasiness or nervousness usually over an impending or anticipated ill*,” a concept far from fitting the problem of anxiety in dentistry. A better definition is available in Oxford Medicine Online^2^: “*Anxiety refers to multiple mental and physiological phenomena, including a person's conscious state of worry over a future unwanted event, or fear of an actual situation. Anxiety and fear are closely related. Some scholars view anxiety as a uniquely human emotion and fear as common to non-human species. Another distinction often made between fear and anxiety is that fear is an adaptive response to realistic threat, whereas anxiety is a diffuse emotion, sometimes an unreasonable or excessive reaction to current or future perceived threat.”*

The Diagnostic and Statistical Manual of Mental Disorders, Fifth Edition (DSM-5) (American Psychiatric Association, 2013) provides a detailed classification of anxiety disorders (Table 1). All anxiety disorders share elements of fear and anxiety, where the former is an emotional response to a real or perceived threat, while the latter concerns expectations of a future threat. Anxiety is a very common disorder with a lifetime prevalence of ~30% (Kessler et al., 2005). About one in two individuals diagnosed with an anxiety disorder also meet the criteria for a depressive disorder (Batelaan et al., 2012). As a result, anxiety in dentistry has two implications in routine clinical practice: (a) the high prevalence of anxiety disorders and depression in the general population, which may make patients anxious during dental care as a result of trait anxiety; (b) a high prevalence of specific dental anxiety (DA) and phobia (i.e., raising up only in the context of dental care), which has been estimated to affect from 10 to 30% of the population, depending on sample selection (i.e., general population or patients scheduled for intervention), ethnic and sociocultural variables (Facco et al., 2008, 2015a). Given the high prevalence of anxiety as a whole, dentists must deal with the phenomenon and its adverse effects in their everyday clinical practice. That is why such European documents and rules as the “*Profile of Competence of the European Dentist*,” published by the Association for Dental Education in Europe (www.ADEE.org), establish that dentists must be competent in identifying, assessing and treating anxiety with both pharmacological and behavioral techniques.

**Table 1:** Classification of anxiety disorders in DSM 5.

Agoraphobia
Generalized Anxiety Disorder
Panic Disorder
Selective Mutism
Separation Anxiety Disorder
Social Anxiety Disorder
Specific Phobia
Substance Induced Anxiety Disorder
Anxiety Disorder Due to Another Medical Condition
Other Specified Anxiety Disorder
Unspecified Anxiety Disorder

## Pathophysiology and assessment of dental anxiety

Given its high prevalence worldwide, dental fear can be considered a universal phenomenon with different cultural features (Berggren et al., 2000). The first reports on the pathophysiology of DA date back to mid-twentieth century with the seminal papers by Coriat (1946) and Shoben and Borland (1954), followed by Forgione and Clark (1974) and Freeman (1985); in the same years, the DA Scale (CDAS) has been developed by Corah (Corah, 1969; Corah et al., 1978) as well as the Visual Analog Scale (VAS), introduced by Aitken to assess emotions and feelings (Aitken, 1969). The interest in pathophysiology and assessment of DA has been paralleled by the first attempts to manage it with both pharmacological approaches (i.e., sedation and general anesthesia) (Goulding et al., 1957; Springer, 1962; Chambiras, 1969; Newman et al., 1970; Machen et al., 1977), and behavioral techniques (Friedman, 1967, 1983; Gatchel, 1980), including hypnosis (Marcuse, 1947; Eycleshimer, 1949; Moss, 1949, 1950, 1951; Kline, 1957; Bertolini, 1970). Since then, an ever increasing number of studies on DA has been published: 878 papers including the words *dental anxiety* or *fear* in the title and 1558 including the same words in the abstract are now available in PubMed, being evidence of the relevance and complexity of the topic.

Coriat emphasized the concept of fear as a form of anticipatory anxiety, not necessarily depending on expected pain (Coriat, 1946); he also defined the fear of a danger which is unknown as a *neurotic anxiety*, related to a feeling of helplessness in an anticipated traumatic situation. Shoben and Borland (1954) investigated the etiology of DA in two groups of 15 patients (anxious vs. non-anxious) checking 11 possible factors, including previous dental or medical bad experiences, previous facial injuries, negative family dental experience or attitude toward dentistry, high anxiety level, dependency, emphasis on appearance or on orality (according to psychoanalysis); the only two factors significantly related to dental fear were the family related ones, leading the authors to conclude that they were the most important factors in determining DA and avoidance behavior. However, these results, though correct, underestimated the role of other factors, such as previous bad experiences, due to the small sample size.

The wealth of data now available makes it definitively clear that the origin of DA is multidimensional and includes both endogenous and exogenous causes (Liddell and Locker, 2000). Several psychological disorders (such as low self-esteem, general fearfulness, conduct disorder, agoraphobia, simple phobia, alcohol dependence, or multiple DMS 5 diagnoses) are more frequent in patients with high DA, as defined by CDAS (Locker et al., 2001; Kvale et al., 2002; Locker, 2003). The exogenous factors include conditioned fear (yielded by previous bad experiences or information), fear of somatic intraoperative reactions and distrust of dental professionals; the latter, in turn, is usually caused by dentists' and/or physicians' inappropriate behavior and traumatic dental treatments, leading to patient's helplessness, threat for autonomy loss and violation (Abrahamsson et al., 2002). Finally, patients with severe systemic diseases may have a higher level of dental anxiety, related to previous experience with their diseases and interventions (Facco et al., 2008, 2015b).

In short, DA is far from being a simple monomorphic clinical entity, where people with no dental fear may have had negative dental experiences, and, vice versa, some people with DA or phobia may fail to recall any traumatic incidents; this calls for a proper understanding of each individual subject, in order to assess the factors involved in his/her DA, be they endogenous and/or exogenous, directly learned from previous bad experiences and/or trough communication with others.

The assessment of the intensity of DA is an essential aspect of patient's evaluation. The estimation of the prevalence of DA may also be affected by the used test to check it; in fact, different anxiety tests may provide different results according to their structure and aims [see Newton (Newton and Buck, 2000) as a review of all main DA tests]. For example the Humphris' Modified Dental Anxiety Scale (MDAS) may improve evaluation of DA when compared to the CDAS, by adding a specific question on dental anesthesia, which is a relevant source for anxiety (Humphris et al., 1995, 2000).

The VAS for anxiety (VAS-A) has proved to be very effective and closely correlated to CDAS, MDAS and Spielberger's State Trait Anxiety Inventory (STAI), but a discordance rate of 25–30% has been detected, suggesting a higher sensitivity of VAS-A (Facco et al., 2011b, 2013b). This discordance probably depends on the different aims of these tests, providing different information: (a) CDAS and MDAS detect DA components related to the dental setting; (b) the STAI- form Y1 detects *state* anxiety (i.e., the anxiety the subject feels when filling in the form), while the STAI-Y2 detects the *trait* anxiety, (i.e., the anxiety perceived in everyday life); (c) the VAS-A, being a non-verbal test, provides an overall estimation of patient's anxiety (not limited by scenarios), and, when administered during the preoperative visit, provides information on patient's DA when reckoning with undergoing surgery (Facco et al., 2013b).

The above mentioned features may explain discordant cases, such as patients with low CDAS and STAI and high VAS-A, who are neither anxious nor fearful of the dentist, but are facing the operation with a strong fear of its possible consequences (e.g., the informed risk of possible inferior alveolar nerve lesions during wisdom teeth removal). Therefore, in our department we decided to routinely use both VAS-A and MDAS and considered as anxious all patients with a high score of at least one test.

Another major source of variability of DA estimation depends on the grading of test scores. In the literature, CDAS, MDAS, and VAS-A have been graded into the following three levels: (1) Not anxious (CDAS<12, MDAS<14, VAS-A<51 mm); (2) anxious (CDAS = 12–15, MDAS = 14–18, VAS-A = 51–75 mm); and phobic (CDAS>15, MDAS>18, VAS-A>75 mm). In all these test the threshold for DA has been set at the mid value of each scale, to be regarded as the threshold for a clinically relevant anxiety. Generally speaking, any limit, despite reliably identified, is somehow arbitrary: in the case of DA tests it does not mean that patients slightly below the established threshold are not anxious. Therefore, the reported prevalence of anxiety in population, despite remaining a valid estimation, is partly conventional and one should be aware that several patients with a score below the mentioned limits may be anxious enough as to deserve its management.

Given the wide range of causes, DA should be regarded as part of the anxiety disorders included in the DSM 5, rather than as separate entities confined to the dental setting (Berggren et al., 2000; Facco et al., 2015b). Among them, previous distressing experiences remain a major cause of anxiety and phobia and share several features with panic attacks and post-traumatic stress disorder (PTSD) (de Jongh et al., 2006). In a series of 230 patients (Facco et al., 2015b), 106 (46%) reported previous bad experiences in dental and/or medical settings and those with bad experiences in both had significantly higher MDAS score than people without bad experiences (17.4 ± 5.2 vs. 11.3 ± 4.5); among 83 (36.1%) attending dental visits only when painful or in trouble, 51 (61.4%) reported such bad experiences. This suggests that the avoidance behavior is related to previous traumatic experience in the majority of cases, while the remaining ones depended on other factors, like DA due to other causes, cultural factors or the barrier yielded by costs (Armfield and Ketting, 2015).

The above data show the crucial role played by health professionals in the pathophysiology of DA, by leading to patients feeling unbearably helpless, being threatened with the loss of their autonomy, and violated, yielding avoidable suffering, pain, and opening the doors to medical emergencies. Thus, dentists and physicians look like the two-faced Janus, the Ancient Roman god of time in the past and future, in war and peace (the month of January is named after him): they may be gentle and protective experts providing safe and painless care, or turn into torturers capable of causing great suffering. Their inappropriate behavior can exacerbate existing anxiety disorders or trigger a new form of anguish (anxiety, phobia, and PTSD) that may persist for life in not properly managed.

The fear they may induce throws us back to ancient times, when our ancestors had to face and react to dangers and predators on a daily basis.

In short, DA can be seen as a complex response of the modern human's mind-body unit (MBU) to a wide range of factors (Bracken, 2002); anxiety and pain during dental treatment may also trigger physical changes, which belong to the so-called *fight-or-flight* reaction and may give raise to emergencies. The incidence of medical emergencies in the dental setting is not rare and ranges between 0.7 and 10 cases/dentist/year; most of them are not disease-related, with vasodepressor syncope being the most frequent one, followed by hortostatic hypotension, hypertension and hyperventilation (Matsuura, 1989; Niwa et al., 1996; Arsati et al., 2010).

It is worth recalling that anxiety and depression had been recognized as inseparable psychosomatic phenomena already in antiquity: in the second century AC, Galen of Pergamon named them *Melancholia hypochondriaca* (from the Greek μ$\overset{´}{\varepsilon }$λας *mélas, black*, and χ*o*λ$\overset{´}{\eta }$, *cholé, bile*) to underscore their physical origin or manifestation (e.g., neurovegetative changes) at visceral level (in the liver, according to Galen).

The complex interplay of the multiple aspects of anxiety can be usefully discussed in terms of “*set and setting*” (Leary et al., 1969; Zinberg, 1986; Hartogsohn, 2013), where set indicates the particular *mindset* of the subject (i.e., mental state, thoughts, expectations, intentions, preparedness for particular experiences, personality structure, mood at the time, etc.), and setting denotes the physical environment (weather, light or dark, indoor or outdoor, etc.) and sociocultural features (values, social structure, and culture). Set and setting are clearly shaped by the particular sociocultural *paradigm* in which a given experience takes place (Kuhn, 1970), so we can differentiate between two levels of set and setting, one individual and the other collective, that are intimately connected and influence one another. Set and setting seem to reflect a uniquely human experience strongly embedded in, and entangled with human consciousness and social life. In the present context, set and setting are represented by the patients/MBUs sitting in the dental chair (and their parents in the case of children), and the key issue concerns how the professionals taking care of them can best manage their problems.

## The birth of anxiety (and dentistry)

The long journey in the evolution of consciousness began millions of years ago, passing through the *genus Homo* to the *Homo sapiens species*, and to the modern *Homo sapiens sapiens*, and it may continue toward a form of *cyborg* or *fyborg* (Warwick, 2003; Kurzweil, 2005). Along the way, the *great change* in human consciousness and, arguably, in humans' emotions and anxieties, probably started some 100,000 years ago, with an event that some archeologists have named the *Sapient Paradox* (Mellars, 1991; Ramachandran, 2000; Iacoboni et al., 2005; Gabora, 2007; Renfrew, 2008; Richerson et al., 2010; Sterelny, 2011; Abramiuk, 2012; Garofoli and Noel Haidle, 2014).

The paradox stems from the observation that our DNA was much the same across the ages since 100,000 years ago, while the explosion of human culture only dates from about 20,000 years ago, anticipated by cave paintings in France and Spain (dating back to about 30,000 years ago). It is hard to explain this time lag on genetic grounds only (Richerson et al., 2010).

The sapient paradox has its critics (Gabora, 2007; Abramiuk, 2012; Garofoli and Noel Haidle, 2014), however, since it relies on an over-simple relationship between genome, mind and culture. The topic has been investigated by cognitive archeologists too, who combine archeology and the neurosciences to study the evolution of consciousness in the genus *Homo*, and particularly in *Homo sapiens*. Their aim is to glean information on how ancient people were thinking and feeling when they built and used the objects that have been found, and to shed light on their very human features, such as perceptions, emotions, attribution of symbolic meanings, mental processes of comprehension, planning, decision-making, communication, and education.

A heated debate has developed between mainstream and cognitive anthropologists, the former convinced that archeological records can only preserve people's actions, the latter claiming instead that such actions are the result of human thought, guided by experience and beliefs. The topic seems to be generally governed by the debate between mechanist-reductionists and non-reductionists, like the controversy on the science of consciousness (Facco et al., 2015a, 2017). Deliberately or serendipitously created, ancient artifacts demonstrate an in-depth knowledge and the acquisition of skills. Studying them can provide us with important information on the consciousness, thoughts and beliefs of our ancestors, shedding light on their *Weltanshauung* (*worldview*) and the so-called, ever-changing *Zeitgeist* (*spirit of the times*) that Jung set against what he called the *spirit of the depths*^3^.

Whatever it may be, Jung's *spirit of the depths* relates to a set of psychological resources (e.g., unconscious thought, motivation and creativity) capable of overriding a given *Zeitgeist* and *Weltanshauung*, and probably reflecting the non-genetic factors most likely involved in the *Sapient Paradox*. Though it may appear philosophical, this approach is pragmatic and closely linked to both archeology and the neurosciences. It lies at the very heart of archeological science, dealing with our knowledge of the behavioral processes of *H. sapiens*, and the sociocultural aspects of his life that we can glean from the archeological records.

A theory has been advanced that *Hominini* underwent a concomitant evolution in tool-making and language. In other words, tool-making, social living and learning, and culture were interactive in our genetic background and essential for human evolution, adaptation and survival (Laland et al., 2010; Boyd et al., 2011; Sterelny, 2012; Morgan et al., 2015). Since different *species* (i.e., *Neanderthalensis, Heidelbergensis*, and *Sapiens*) of the genus *Homo* lived as neighbors on this planet for long periods of time, they probably imitated one another, facilitated by the mirror neuron system (Ramachandran, 2000; Rizzolatti and Craighero, 2004; Iacoboni et al., 2005; Filimon et al., 2007), learning behaviors, attitudes, and rituals useful for survival (Wrangham and Carmody, 2010; Potts, 2013; Parravicini and Pievani, 2016). Archeological records also provide some evidence of very human behavior in the management of diseases and injuries (e.g., amputations, repair of severe long bone, and skull fractures), congenital skeleton deformities (e.g., dwarfism), hydrocephaly, etc. (Trinkaus and Zimmerman, 1982; Lordkipanidze et al., 2005; Buquet-Marcon et al., 2007; Oxenham et al., 2009). The severely disabled received long-term care in Neolithic times, as in the reported case of a patient with Klippel-Feil Syndrome (a congenital fusion of the spine) who survived for more than 10 years, despite depending on others for his survival (Oxenham et al., 2009). A case of an early *Homo* (dating back to 1.77 million years ago) who had lost all but one tooth several years before his death also makes us wonder about his alternative subsistence strategies, which may have included receiving help from other individuals (Lordkipanidze et al., 2005). On the whole, there is a growing body of evidence that these ancient humans experienced some degree of consciousness as well as emotions, exploring their surrounding natural environment and seeking help and solutions to their daily problems (Dettwyler, 1991; Humphrey, 1998; Cross, 1999; Tarlow, 2000; Spikins et al., 2010; Apicella et al., 2012; Hardy et al., 2012, 2013; Tilley, 2015).

Archeological records, dating back to the Late Upper Paleolithic Era, provide evidence of a kind of “*proto-dentistry*,” revealing a surprising amount of knowledge and skill (Irish, 2004; Coppa et al., 2006; Bernardini et al., 2012; Gutmann, 2015; Oxilia et al., 2015). They include teeth showing signs of drilling and filling with beeswax, as well as carvings on the cavity wall with a micro-tool, probably for therapeutic-palliative purposes. Archeologists were able to rebuild a bow-drill with a flint head, required to produce such holes in human enamel. Perhaps this mastery was originally developed by skilled artisans for the purpose of making beads, and was subsequently transferred to the drilling of teeth. Furthermore, several ancient peoples (e.g., Mesoamerican Mayas and Vikings) practiced a manual sharpening of the teeth, especially of the front incisors, the purpose of which was cultural rather than medical (De Mello, 2007)^4^,^5^.

Judging from the above-mentioned data, it seems reasonable to argue that forms of human anxiety—including DA—can be seen as a sort of odyssey begun in prehistory, when our ancestors were faced many times a day with danger, pain, stress, and the related *fight-or-flight* responses, as well as with diseases, toothache and some sort of dental treatments. Animals clearly know fear too, but human beings are often reluctant to admit that they belong to the animal kingdom (Panksepp et al., 2012). On the other hand, human anxiety also entails a much greater use of memory and imagination with respect to animals, enabling one to move backwards and forwards in time, a mind faculty related to the human's well-developed default mode network (Buckner et al., 2008; Andrews-Hanna et al., 2010).

## Anxiety and pain: an indissoluble marriage

According to the *fight-or-flight* theory, our first reaction to danger involves a sympathetic activation that can be regarded as the early stage of a general adaptation syndrome adopted by many animals, including *H. sapiens*. This acute stress response—described many years ago by Bernard (1973) and Cannon (1929a,b, 1932)—involves several organs and systems (including the central and peripheral nervous system, the cardiovascular apparatus, the endocrine and immune systems, and the skeletal muscle), and yields specific physiological and psychosomatic changes through the release of several chemical mediators and neurotransmitters (e.g., epinephrine and norepinephrine). The resulting neuro-immune-endocrine storm triggers the well-known *centralization* of the circulation (vasoconstriction, and an increase in blood pressure, heart rate and respiratory rate) in order to assure a sufficient blood supply to the heart, brain, lungs, and skeletal muscles, and thus facilitate actions needed in fighting or fleeing. This reflects the situation experienced by patients in the dental chair when they feel threatened by the dentist, making the latter resemble the enemy and predator, or even the proto-dentist of ancient times.

The concept of *fight-or-flight* has evolved into a more structured theory, better fitting the complexity of stress reactions, called the *Polyvagal Theory*, which was introduced by Porges at the end of the last century and subsequently expanded by other authors (Porges, 1995, 2003, 2004; West, 2010; Quintana et al., 2012). According to the polyvagal theory, social and defensive behaviors in mammals, and primates especially, are controlled by particular brain structures and circuits that have evolved over a very long time into three different stages: *immobilization, mobilization*, and *social engagement*. With evolution, the stress response has developed into a broader range of behaviors: fighting may now be in the form of expressing anger and quarreling, while flight may take the shape of a vasodepressor syncope, the most common medical emergency in the dental office (Malamed, 1997). The latter has become more common today because stress responses are usually prompted in settings (such as the dental office) where fighting is considered inappropriate for well-educated adults. It is worth noting that children are less likely to experience any vasodepressor syncope because they are more likely to fight against the dentist if necessary, thus closing the physiological loop of their reaction.

The polyvagal theory has brought with it the novel idea of *neuroception* (Porges, 2004), which describes how human beings can distinguish between safe and dangerous or life-threatening situations. This ability of our consciousness still triggers certain neurobiologically-determined prosocial or defensive responses depending on the perceived context, and explains why we may react differently in similar settings (an infant may coo at a caregiver, but cry at a stranger, for instance). Some patients may experience DA or phobia as a result of several causes, such as earlier unpleasant experiences in medical/dental settings, or relationships with a dentist (Facco et al., 2008, 2015a).

Pain is the other essential factor related to anxiety and emergencies in the dental office and, as easily understandable, the most feared intervention are root canal and restorative treatments without local anesthesia, as well as oral surgery (Collado et al., 2008).

Pain is a universal phenomenon, the symptom *par excellence*, and a major health problem the world over, severely affecting people's overall quality of life. The International Association for the Study of Pain (IASP) has defined pain as “*An unpleasant sensory and emotional experience associated with actual or potential tissue damage, or described in terms of such damage.”* (Bonica, 1979; Merskey, 2008)^6^. This definition avoids tying pain to nociceptive pathways alone, and clearly shows that its nature is essentially a matter of experience. The full definition was accompanied by a note which is worth including here to properly understand its nature: “*The inability to communicate verbally does not negate the possibility that an individual is experiencing pain and is in need of appropriate pain-relieving treatment. Pain is always subjective. Each individual learns the application of the word through experiences related to injury in early life. Biologists recognize that those stimuli which cause pain are liable to damage tissue. Accordingly, pain is that experience we associate with actual or potential tissue damage. It is unquestionably a sensation in a part or parts of the body, but it is also always unpleasant and therefore also an emotional experience…Many people report pain in the absence of tissue damage or any likely pathophysiological cause; usually this happens for psychological reasons* [or due to unrecognized functional causes; Authors'note] …*If they regard their experience as pain, and if they report it in the same ways as pain caused by tissue damage, it should be accepted as pain. This definition avoids tying pain to the stimulus. Activity induced in the nociceptor and nociceptive pathways by a noxious stimulus is not pain, which is always a psychological state, even though we may well-appreciate that pain most often has a proximate physical cause.”*

Although pain is subjective, a matter of experience and emotion, most of research and clinical practice have only focused mechanistically on analgesic and anesthetic drugs, understating its nature and forgetting the patient's role in its management (Hill, 2006; Aydede, 2009; O'Sullivan and Schroer, 2012). In short, pain is a matter of experience, a subjective psychological state that does not necessarily have a detectable organic cause. It is a complex functional phenomenon that depends on a wealth of factors and can be classified in various ways, as acute, chronic, incident, procedural, etc. The procedural pain perceived by patients undergoing medical/dental procedures is an important and common cause of anxiety, stress, *fight-or-flight* reactions, and vasodepressor syncope. Anxiety and pain are thus two partners that have always existed in real-life, as well as in dental and medical care, and that have always had the potential for turning into a vicious circle.

## History and the role of pharmacological techniques today

The first documented evidence of human beings using medicinal herbs dates back to *Homo Neanderthalensis* (around 60,000 years ago), when small groups of hunter-gatherers were wandering all over the planet, encountering very different territories and landscapes, and carving out ecological niches for their self-preservation (Sommer, 1999; Hardy et al., 2012, 2013; Zorich, 2012). Hardy and Buckley examined the chemicals embedded in the calcified plaque on the teeth of five *H. Neanderthalensis*, from the El Sidrón Cave in Spain (Hardy et al., 2012, 2013). They found that *H. neanderthalensis* cooked and ate plants, including bitter-tasting medicinal ones like *Matricaria chamomilla* and *Achillea millefolium*.

It is well-recognized that the perception of a bitter taste is useful for survival because it can regulate the intake of foods containing toxic substances, and prevent poisoning, making it easier to adapt especially in wild environments. Bitter taste perception is mediated by G-protein-coupled receptors, expressed in taste cells on the surface of the tongue and encoded by the TAS2R gene family, which was also present in *H. neanderthalensis* (Lalueza-Fox et al., 2009). Interestingly, both the above-mentioned plants have little nutritional value, but are well-known for their medicinal qualities and still used today for their anti-inflammatory and sedative properties, in teething toddlers, for instance, and in insomnia and anxiety disorders.

Our ancestors observed and mimicked wild and domestic animals, and this helped them to identify herbal remedies and foods. To give an example, both chimpanzees and *H. sapiens* in sub-Saharan Africa are known to ingest the bitter pith of *Vernonia amygdalina* to control intestinal nematode infections (Huffman, 2003). There is also widespread evidence of self-medication in animals, defined zoo-pharmacognosy, which helps to explain the use of medicinal plants by many species of *Homo* in bygone times (Etkin and Ross, 1982; Huffman, 2001, 2002; Rodriguez and Wrangham, 1993). Animals and humans also seem inclined to ingest, even to excess, foodstuffs with mind-altering properties. It is well-known that reindeer (but also bears and other animals) enjoy the hallucinogenic mushroom *Amanita muscaria*, which makes them behave as if they were drunk and then fall asleep. On waking, reindeer even urinate and then devour the urine-soaked snow to perpetuate their intoxication (Dobkin de Rios, 1984a; Samorini, 2002; Engel, 2003). Siberian tribespeople like the Chukchee, Koryak, Kamchadal, learned from the reindeer and, ever since ancient times, their shamans used *A. muscaria* to induce a state of *trance* or *ecstasy* in order to communicate with the supernatural, divine the weather, diagnose and treat disease, including psychological and psychosomatic disorders (Dunn, 1973; Dobkin de Rios, 1984b).

A discussion about shamanism, defined by Eliade (1972) as the “*archaic techniques of ecstasy*,” is well-beyond the scope of this article (for further details, see Huffmant, 1983; Bednarik, 1990; Lewis-Williams and Dowson, 1993; Lewis-Williams and Clottes, 1998; De Lumley, 2002; Navet, 2002; Van Pool, 2002; Lewis-Williams, 2004; Aldhouse-Green and Aldhouse-Green, 2005; Devereux, 2008; Henshilwood, 2009), but it is worth noting here that shamanism may be understood as a complex knowledge/belief system developed over thousands of years, in an attempt to control the natural world and disease. Etymologically, the term “*shaman*” means “*man of knowledge”* and, since knowledge means power, “*man of power*”; this is fully consistent with Francis Bacon's definition of science as power, and with the idea of the history of modern science as the history of the will to power (Severino, 1980).

Among hundreds of natural products used by the shamans, the most powerful were the psychotropic plants, which were capable of taking humans to realms of ethereal wonder. These plants, called *plants of power, plants of knowledge*, or *plants of the gods*, were used to manage people's problems, help them adapt to stress and adversity, and promote resilience (the main plants used by our ancestors and by contemporary native populations are summarized in Table 2 (Von Bibra, 1855; Cooke, 1860; Mantegazza, 1871; Lewin, 1927; Farb, 1968; Bourguignon and Evascu, 1977; Dobkin de Rios, 1984c; McKenna, 1992; Samorini, 1992; Schultes and Hofmann, 1992; Zias et al., 1993; Zias, 1995; Ott, 1997; Bruhn et al., 2002; Donald, 2003; El-Seedi et al., 2005; Ratsch, 2005; Balick et al., 2007; Russo et al., 2008; Akers et al., 2011; Gosso and Webster, 2013; Guerra-Doce, 2015). What is intriguing is their use on the grounds of a valuable intuition, exploiting their therapeutic effects in several disorders (including pain, anxiety, insomnia, PTSD, migraine, nausea and vomiting, menstrual and gastrointestinal upset, drug addiction, and epilepsy), even with no knowledge of their chemical composition. Like the efficacy of many of these traditional remedies, their composition has been established by modern science (Schultes, 1972; Nichols, 1999; Roberts, 1999; Griffiths et al., 2011; MacLean et al., 2011; Winkelman, 2014).

**Table 2:** Some of the most popular medicinal plants provided with psychotropic effects and their chemical derivatives used in traditional medicine all over the world.

**Botanical name**	**Nick name**	**Main chemicals contents**
*Amanita muscaria*	Fly Agaric	Ibotenic acid, muscimole
*Psilocybe mexicana*	Magic Mushroom	Psylocibine, psilocine
*Lophophora williamsii*	Pejote	Mescaline
*Salvia divinorum*	Salvia	Salvinorine
*Banisteriopsis caapi*	Ayahuasca	Tryptamines
*Datura stramonium*	Thorn apple	Tropane alkaloids
*Tabernante iboga*	Eboka	Ibogaine
*Erythroxylum coca*	Coca	Cocaine
*Papaver somniferum*	Opium	Opioids
*Cannabis sativa*	Marihuana	Phyto-cannabinoids
*Nicotiana tabacum*	Many	Nicotine
*Ethyl alcohol*	Many	Ethanol

With time, medicine underwent a huge evolution, first in ancient Egypt and then in Greece. The Edwin Smith Surgical Papyrus (dating back to 2,500–3,000 years BC) describes 48 clinical cases of neurological injuries and related brain lesions (e.g., aphasia and hemiplegia), giving detailed accounts of the brain's anatomical features, including the cranial sutures, meninx, and cerebrospinal fluid (Cunha, 1949; Helgason, 1987; Minagar et al., 2003; Cosmacini, 2015). As for pharmacological anesthesia, the so-called *Aleppo sponge* (steeped with a mixture of opium, cannabis, hyoscyamus, mandragora, black nightshade, and other plants containing tropane alkaloids) was used by ancient Arabian physicians and other populations to induce sedation and a sort of general inhalational anesthesia (the sponge was placed over the patient's nose/mouth; Ajram, 1993; Hehmeyer and Khan, 2007). In South America, the Maya, and Inca also used plants with tropane alkaloids to induce sedation, as well as *Erythroxylum coca* leaf extract as a kind of local anesthetic (Fairley, 2007; Stolberg, 2011; Biondich and Joslin, 2016).

The above data suggest an intriguing functional connection between plants and the nervous system of animals (*H. sapiens* included) mediated by phytochemical compounds (Dobkin de Rios, 1984c; McKenna, 1992; Schultes and Hofmann, 1992; Ott, 1997; Ratsch, 2005; Dawson, 2009; Krippner and Luke, 2009; Mancuso and Viola, 2015), such as the phyto/endo-cannabinoid system, the phyto/endo-opioid system, or the phyto/endo-tryptamine system (Vogel et al., 1993; Di Marzo, 2009; Benarroch, 2012; Araújo et al., 2015). This ages-old bond between plants and animals has been essential to the survival, evolution and adaptation of the genus *Homo*. It may even have helped to drive the *great change*, or the *sapient paradox*, during the Neolithic age and the development of our particular human features (McKenna, 1992; Ott, 1997; Strubelt and Maas, 2008; Campos et al., 2012, 2013; Ganon-Elazar and Akirav, 2013). It shows the intimate connectedness between the plant and animal kingdoms in an inseparable whole (Stephenson, 2012; Harwood and Ruuska, 2013; Laughlin, 2013), where animals help plants to survive by spreading their seeds, and plants help animals to adapt, providing them with nourishment, medicines and psychotropic effects that have accompanied the spiritual evolution of humanity since prehistory through the Eleusis Mysteries of ancient Greece up to the native populations of the present day (Wasson et al., 1978; Samorini, 2000; Keller, 2009).

The stressful scenario of dentistry demands an appropriate management of anxiety, pain and the related physical reactions in order to improve the overall safety of dental care, and make the patient's centered approach the ethical gold standard in modern dentistry. Pharmacological anxiolysis is a keystone of this fundamental goal. To be effective, anxiolysis must be integrated with an effective local anesthesia, able to prevent pain during dental procedures. The whole of anxiolysis and local anesthesia is a complex topic, far beyond the aim of this article; here, it is only worth stressing that two different approaches have been adopted in North American and European Countries. The North American stance is based on techniques of deep sedation and general anesthesia, involving the use of many different sedative/anesthetic drugs with their possible combinations (American Dental Association, 2016). Moreover, physical restraint and hand-over-mouth techniques have been largely admitted and accepted by parents in struggling children until now (Wilson and Houpt, 2016). Unfortunately, they have not been abandoned yet, despite the increasing distrust toward these physical methods of immobilization and coercion (Newton et al., 2004; Eaton et al., 2005); this is no longer tenable, given that previous bad experiences in the dental setting are a major cause for DA and phobia, with their wide range impact in patients' lives (Levin, 2003; Facco et al., 2015b).

Unlike US, European stance holds anxiolysis and conscious sedation, which have been defined as follows (Report of the Intercollegiate Advisory Committee for Sedation in Dentistry, 2015): “*A technique in which the use of a drug or drugs produces a state of depression of the central nervous system enabling treatment to be carried out, but during which verbal contact with the patient is maintained throughout the period of sedation. The drugs and techniques used to provide conscious sedation for dental treatment should carry a margin of safety wide enough to render loss of consciousness unlikely.”*

In other words, the sedation's level must be such that the patient remains conscious, with fully preserved vital functions, and able to both understand and respond to verbal commands, either alone or accompanied by a light tactile stimulation. In the continuum from the fully conscious state to general anesthesia, a progression beyond the level of conscious sedation must be considered to be deep sedation or general anesthesia. Given the impairment of protective reflexes yielded by deep sedation and the increased likelihood of adverse events, it is subject to different regulations and calls for an escalation in the competency required to ensure safe sedation practice (Zanette et al., 2007; Pandit, 2014).

The European perspective considers the intravenous or inhalational administration of a single sedative drug (benzodiazepine and nitrous oxide are the preferred ones, respectively) and the endpoint is reached by titration (i.e., the administration of incremental doses) until a full anxiolysis is reached with preserved consciousness; physical restraint is not allowed in Europe and may elicit legal claims (Zanette et al., 2007). The rationale for the European recommendations is that the target is the withdrawal of anxiety and pain: that's all, and can be fully achieved with conscious sedation and local anesthesia. Avoiding the use of hypnotic drugs, opioids, and general anesthetics, allows for several relevant advantages: (a) patient's full tranquility and collaboration are assured, easing the dentist job; (b) there is neither risk of inhalation of saliva, blood and debris, nor need of tracheal intubation to control the airway; (c) high simplicity and safety, which make conscious sedation a technique easily managed by the dentist; (d) no risk of major anesthesia related complications.

In conclusion, the European recommendations regarding conscious sedation, should be regarded as the safest and simplest way of managing the patient, able to improve safety through emergency prevention rather than being a cause of adverse events, while the use of deep sedation and general anesthesia should be indicated in selected, non-collaborating patients only. A routine use of deep sedation and general anesthesia is affected by a higher risk of severe complication and deaths, a fact and a concern of US dentists and insurances (Yagiela, 2001; Chicka et al., 2012; Lee et al., 2013; Reuter et al., 2017). In our experience, we performed more than 15,000 intravenous conscious sedations using our dedicated protocol, with no complications at all (1,179 cases have been published elsewhere, Manani et al., 2005).

## Prevention of anxiety, pain and emergencies in dentistry: behavioral techniques

In ancient Egyptian and in Greek medicine, along with the knowledge of medicinal plants and development of surgical procedures came the use of incubation, a healing technique that can be regarded as the ancestor of modern hypnosis. Incubation was a healing technique practiced in Egyptian medicine as part of the cult of Isis at Memphis, Serapis at Canope, Alexandria and Thebes (Cosmacini, 2015). It was also used in Greece, since before Homer's time, in the temples of Apollo and Asclepius, which were also called S*leep Temples*. Parmenides, the great pre-Socratic philosopher well-known for his definition of *Being and Nihil*, was also a priest of Apollo, *iatromantis* (healer) and expert in incubation (Kingsley, 1999; Facco, 2014), while Hippocrates was an Asclepiad.

Asclepius temples included the ἄβατ*o*ν (à*baton, the inaccessible*), where patients were incubated for a night, during which they were induced to dream of the god, who gave them advice and instructions on how to recover from their illness. Later on, with advances in surgical treatments, incubation as well as plants extracts, like opium, was used in the attempt to enable painless surgery as well. A testimony by Artemidorus [*Onirocritica*, V, 61; quoted by Edelstein (Edelstein and Edelstein, 1998), p. 259] reports about surgery during incubation in a case of abdominal abscess: “*A certain man dreamed that, struck in the belly by Asclepius with a sword, he died; this man, by means of an incision, healed the abscess which had developed in his belly*”.

Hippocrates, the universally recognized founder of the rational medicine, held a holistic approach emphasizing the role of mind in therapy and recovery, as witnessed by Plato in the Phaedrus (p. 270b–e): “[Socrates] *Why, because medicine has to define the nature of the body and rhetoric of the soul-if we would proceed, not empirically but scientifically, in the one case to impart health and strength by giving medicine and food in the other to implant the conviction or virtue which you desire, by the right application of words and training…Hippocrates the Asclepiad says that the nature even of the body can only be understood as a whole”*.

Following the Descartes' radical dualism, splitting the soul and the body, the modern medicine devoted itself to the cure of the Cartesian *earthen machine* only, more and more disregarding consciousness and subjective phenomena and moving through positivism and scientific reductionism, thus, understating the Hippocrates' teaching of the *whole*, and the therapeutic role of *words and training* until the latter twentieth century. In this climate, behaviorism neglected the relevance of consciousness, while hypnosis was misunderstood and rejected a priori as absurd and, anyway, irrelevant. Instead, the relevance of psychology and behavioral sciences in dental education and clinical practice has progressively increased in the past two decades. Oral surgery is a stressful condition, causing a relevant increase of anxiety, expected suffering and pain perception immediately before the operation (Eli et al., 2000, 2003), while intraoperative anxiety and pain are the main cause for emergency in dentistry and bad oral health, the latter due to delaying or avoiding treatments (Berggren and Linde, 1984; Berggren et al., 2000; Haugejorden and Klock, 2000). Therefore, their assessment and prevention is an essential part of safety and overall quality of care. Anxiety and phobia in turn make the dentist's job hard and stressful (Moore and Brodsgaard, 2001; Hill et al., 2008), a fact leading to the idea, perhaps a myth, of a high suicide rate among professionals in the past years (Stein, 2004; Jones et al., 2016).

Several behavioral techniques allow for a proper patient's management, including the above mentioned use of psychological tests for the assessment of DA (Facco et al., 2008, 2015b), iatrosedation (Friedman, 1967, 1983, 1993; Friedman and Wood, 1998; Taneja, 2015), empathic communication (Wiltshire et al., 2002; Parkin et al., 2014), and hypnosis (Facco et al., 2013a,b, 2014; Facco and Gonella, 2015), but they are still underused, looking traditionally incompatible with the ruling reductionist approach. Of course, pharmacological sedation may be added as well, when needed.

Empathic communication and iatrosedation are the starting point of the approach to the patient, may strongly help relieving anxiety and in several cases these are enough to face the dental care (Friedman, 1967, 1983). Hypnosis has a stronger potential, in that it can be effectively used to decrease or recover from DA in the preoperative phase as well as induce a deep relaxation and increase of pain threshold during dental care; in patients with high hypnotic ability, hypnotic analgesia may also allow to reach the level of surgical analgesia (Casiglia et al., 2007; Facco et al., 2011a, 2013a).

**Figure 1:**
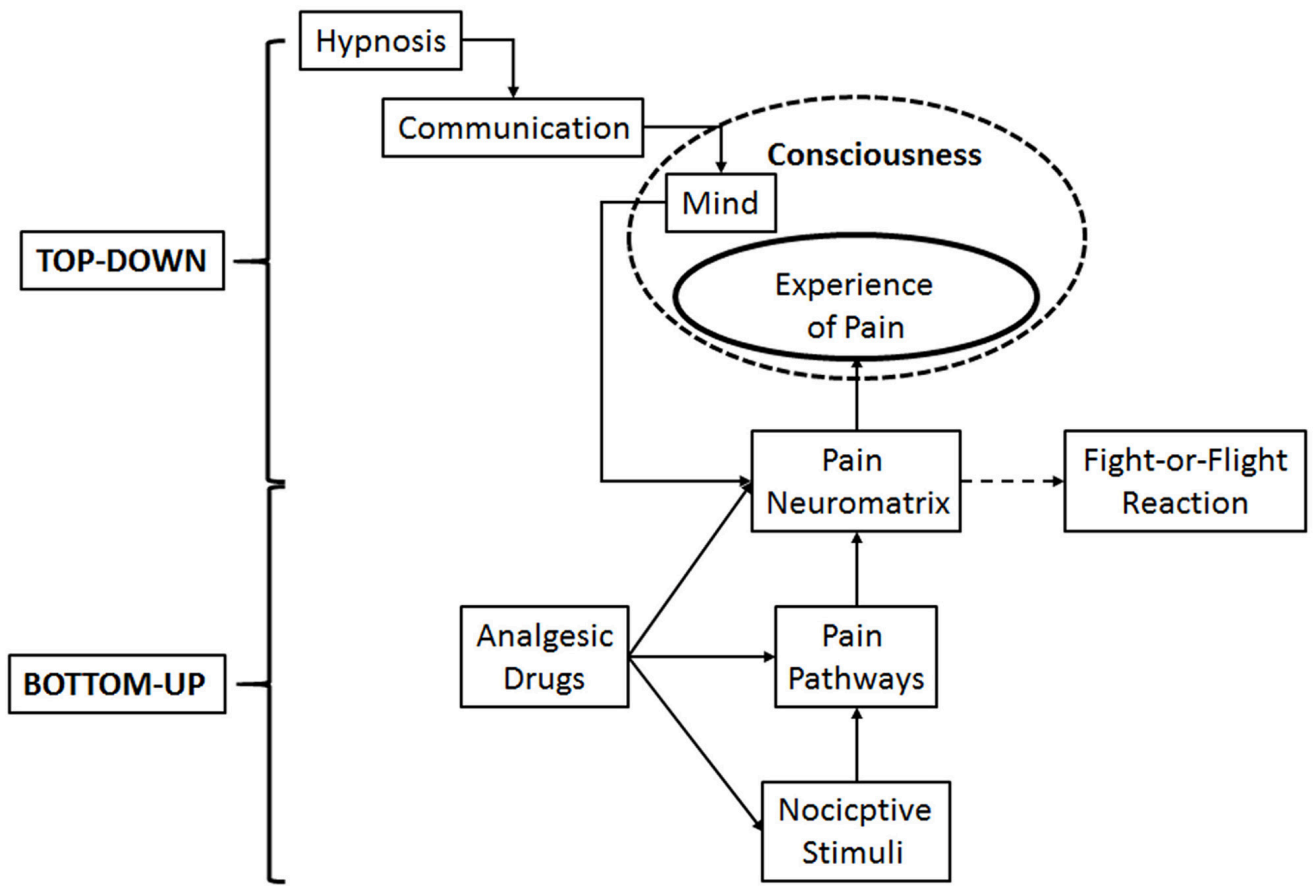
Schematic representation of the double path of pain management: Pharmacological interventions, by modulating the activity of pain pathways, may be regarded as bottom-up procedures, while hypnosis may be regarded as a top-down intervention, by altering anxiety and pain through mental activity on the neuromatrix and limbic system. Both of them modulate the stress response.

Hypnosis has been proven to yield specific changes in several brain areas and circuits, according to the aims of delivered suggestions, such as changes of activation and connectivity of pain neuromatrix, default mode network and extrinsic system (Rainville et al., 1999, 2002; Faymonville et al., 2003; Rainville and Price, 2003; Derbyshire et al., 2004; Roder et al., 2007; Demertzi et al., 2011; Deeley et al., 2012; Vanhaudenhuyse et al., 2014; Facco, 2016). It has proved to be a valuable technique in perioperative care, able to improve recovery after surgery. For the sake of coherence with the very definition of pain, its management should take into account a double path, including both drugs and behavioral techniques able to alter its experience. Analgesic drugs are effective in modulating the activity of pain pathways in the peripheral and central nervous system at different levels with different mechanisms, thus affecting pain perception up to the level of surgical analgesia. Hypnosis may reach the same target by directly modulating pain perception through an introspective mental activity able to change the connectivity of the pain neuromatrix in the brain. Pain neuromatrix, through the anterior cingulate cortex and the connections of the limbic system with the structures responsible for the fight or flight reaction may in turn open the doors to the anxiety and pain related emergencies in the dental setting. Therefore, pharmacological interventions may be regarded as bottom-up procedures, affecting anxiety and pain from pain pathways and limbic system to consciousness, while hypnosis may be regarded as a top-down intervention able to affect anxiety and pain through mental activity on the neuromatrix and limbic system (Figure 1). Therefore, it looks reasonable to speculate that drugs and behavioral techniques might be gathered in a whole, exploiting their different mechanisms in a synergistic way.

Of course, the mentioned behavioral techniques should not be considered as separate, independent tools; rather, they are to be regarded as a continuum in the communication skill of the dentist, since their common relevant tract is the empathic relationship and the capacity of taking care of the patient, instead of teeth only. Pharmacological sedation may be added when behavioral techniques are not enough and remains the essential, valuable technique in non-hypnotizable patients and those with special needs (such as non-collaborating ones). Anyway, as already emphasized by Friedman in his revolutionary approach to the anxious patient fifty years ago (Friedman, 1967), it only affords a temporary respite in order to help the patient coping with single interventions, while behavioral techniques allow many subjects to recover forever from their anxiety and face dental treatments in full autonomy.

In conclusion, DA and its management are far from being a simple matter of choice of the ideal sedative drug, providing all the wished effects without side effects (i.e., a non-existing drug). Instead, they call for a more complex approach, where behavioral techniques have a primary role, but can be usefully implemented by pharmacological sedation as a secondary tool, according to patients' needs, in order to assure to all cases a reasonably pleasant and safe dental care.

## Conclusions

Anxiety, stress, and pain are a universal human problem of all ages and dentistry has been always associated to anxiety and pain, to be regarded as an inseparable couple. Dental care has been introduced since the Neolithic, making it reasonable arguing that the odyssey of DA started in the prehistory. Since then, the evolution of shamanic as well as Egyptian and Greek medicines have paralleled the progress of human culture, improving their approach to diseases, including a wise use of healing techniques, like incubation, medicinal plants, and surgery.

The huge progress of scientific medicine in last century has allowed for an incredible development of pharmacology and surgery, providing more and more powerful therapeutic tools. On the other hand, it is available to some 30% of population only, while traditional medicines still hold a relevant role in the world.

Despite technology has revolutionized the modern dentistry, a look to the prehistory with its proto-dentistry and the use of medicinal plants lets our modern practice appear as an entirely new one and, at the same time, always the same, i.e., treating toothache and tooth pathology by drilling, micro-tool carving and filling, helped by analgesic, sedatives and behavioral techniques (incubation in old ages). On the other hand, following the rationalistic revolution in seventeenth century and the birth of modern sciences, medicine has dealt with the Cartesian *earthen body machine* only, disregarding the role of patient's subjectivity and relegating psychological suffering and pain to pharmacological manipulation only. The perspective of the ruling mechanist-reductionist paradigm has understood Hippocrates as the founder of rational medicine, but both his holistic approach and incubation have been misunderstood and disregarded, while consciousness itself and soul were disregarded in the first half of twentieth century in both psychology and medicine (Facco et al., 2017). Likewise, hypnosis has been misunderstood and prejudicially rejected for over two centuries, due to its ostensible incompatibility with post-Enlightment rationalism and positivism, despite the evidence of its effectiveness in anesthesia (Facco et al., 2012). As a result, a progressive dehumanization of care has occurred in the past decades, heir of logic positivism and materialism, which reduced medicine to its mechanical aspects only. This has also been favored by what Relman, in 1980, named the *New Medical-Industrial Complex* (Relman, 1980), defined as follows: “*A large and growing network of private corporations engaged in the business of supplying health-care services to patients for a profit …It may be more efficient than its non-profit competition, but it creates the problems of overuse and fragmentation of services, overemphasis on technology, creamskimming, and it may also exercise undue influence on national health policy*”.

In this climate, dentistry has become more and more technological, leading professionals to focus on teeth only, rather than patients; accordingly, DA has been mainly faced seeking for the ideal sedative, an illusory target, and the pragmatic but deplorable use of physical restraint and hand-over-mouth techniques in children until recent years.

DA is a huge and complex problem, the management of which is still below the expectations of patients and the abilities of dentists, despite all European documents and rules state the need for their competence in treating anxiety with both behavioral and pharmacological techniques.

DA and phobia are the result of learning with the same pathophysiology of other anxiety disorders, including PTSD (related to bad experiences); thus, their management is a matter of cognition and problem restructuring, not a simple use of sedative, hopefully ideal, drugs. Furthermore, many phobic patients report previous bad experiences in both medicine and dentistry, showing that a relevant cause of DA is the inappropriate behavior of care givers, a powerful nocebo able to engender lifelong severe psychological suffering (Colloca and Finniss, 2012). At the beginning of the third millennium we do believe that this is no longer tenable on both scientific, philosophical and ethical standpoints. As a result, an empathic approach to the patient, the right dentist's behavior, the use of iatrosedation and hypnosis are to be regarded as the first inescapable step of dental care, which may be implemented, when needed, by a wise use of anxiolytic drugs to get a full conscious sedation, while keeping deep sedation and general anesthesia for selected cases only. This the way to get the best overall quality of care, providing a reasonably pleasant dental care, while preventing complications (Brunton, 2012); in fact, the latter mostly spring from controllable iatrogenic factors, i.e., pain and anxiety on one hand, and an unnecessary deep sedation with its implicit harms on the other hand.

The time is now ripe to reappraise the no longer tenable, unyielding materialist-mechanist paradigm of scientific medicine denying the value of patient's subjectivity and rediscover the holistic teaching of Hippocrates; only this may allow to rebuild a patient-centered approach and a veritable, effective doctor-patient relationship, focused on the “*to care*” instead of the “*to cure*” of the illness-centered medicine; as Hippocrates himself taught, “*It is more important to know what sort of person has a disease than to know what sort of disease a person has”*.

## Author contributions

EF and GZ contributed equally to planning and writing of the manuscript; both authors agree to be accountable for the content of the work.

### Conflict of interest statement

The authors declare that the research was conducted in the absence of any commercial or financial relationships that could be construed as a potential conflict of interest.

## Footnotes

^1^http://www.merriam-webster.com/dictionary/anxiety (Accessed February 12, 2017).

^2^http://oxfordmedicine.com/view/10.1093/9780195173642.001.0001/med-9780195173642-chapter-10 (Accessed February 12, 2017).

^3^*If I speak in the spirit of this time, I must say: no one and nothing can justify what I must proclaim to you*. *Justification is superfluous to me, since I have no choice, but I must. I have learned that in addition to the spirit of this time there is still another spirit at work, namely that which rules the depths of everything contemporary*. *The spirit of this time would like to hear of use and value. I also thought this way, and my humanity still thinks this way*. *But that other spirit forces me nevertheless to speak, beyond justification, use, and meaning*. *Filled with human pride and blinded by the presumptuous spirit of the times, I long sought to hold that other spirit away from me. But I did not consider that the spirit of the depths from time immemorial and for all the future possesses a greater power than the spirit of this time, who changes with the generations*. *The spirit of the depths has subjugated all pride and arrogance to the power of judgment. He took away my belief in science, he robbed me of the joy of explaining and ordering things, and he let devotion to the ideals of this time die out in me. He forced me down to the last and simplest things*. *The spirit of the depths took my understanding and all my knowledge and placed them at the service of the inexplicable and the paradoxical. He robbed me of speech and writing for everything that was not in his service, namely the melting together of sense and nonsense, which produces the supreme meaning. But the supreme meaning is the path, the way and the bridge to what is to come. But with this the spirit of our time had almost brought it about that instead of speaking, I was thinking again about reasons and explanations. But the spirit of the depths spoke to me and said: To understand a thing is a bridge and possibility of returning to the path. But to explain a matter is arbitrary and sometimes even murder. Have you counted the murderers among the scholars?” Jung (**2009**)*.

^4^http://news.nationalgeographic.com/news/2006/02/0203_060203_viking_teeth_2.html (Accessed February 15, 2017).

^5^https://en.wikipedia.org/wiki/Human_tooth_sharpening (Accessed February 15, 2017).

^6^http://www.iasp-pain.org/Taxonomy?navItemNumber=576#Pain (accessed February 12, 2017).
